# Electroacupuncture for postoperative pain after nasal endoscopic surgery: study protocol for a pilot randomized controlled trial

**DOI:** 10.1186/s13063-020-4064-2

**Published:** 2020-02-11

**Authors:** Shanshan Li, Qing Zhang, Xuan Yin, Hongyu Yue, Wei Zhang, Lixing Lao, Zhangjin Zhang, Huangan Wu, Shifen Xu

**Affiliations:** 1 Shanghai Municipal Hospital of Traditional Chinese Medicine, Shanghai University of Traditional Chinese Medicine, Shanghai, 200071 China; 2 Shanghai Jingan District Zhabei Central Hospital, Shanghai, 200070 China; 3 Department of Biostatistics, School of Public Health, Fudan University, Shanghai, 200032 China; 4 School of Chinese Medicine, The University of Hong Kong, Hong Kong, China; 5 Virginia University of Integrative Medicine, Fairfax, VA 22031 USA; 6 Shanghai Research Institute of Acupuncture and Meridian, Shanghai University of Traditional Chinese Medicine, Shanghai, 200030 China

**Keywords:** Electroacupuncture, postoperative pain, nasal endoscopic surgery, RCT, randomized controlled trial, clinical trial

## Abstract

**Background:**

Postoperative pain is common after nasal endoscopic surgery. It interferes with the quality of sleep and delays postoperative recovery. Acupuncture is an effective tool for pain management. However, electroacupuncture specifically for the relief of postoperative pain after nasal endoscopic surgery has not yet been studied in a randomized controlled trial.

**Methods/design:**

This randomized sham-controlled patient- and assessor-blind pilot trial has been designed to evaluate the efficacy and safety of electroacupuncture in managing postoperative pain following nasal endoscopic surgery to treat sinusitis due to nasal polyps. Altogether, 30 participants will be randomly allocated to an electroacupuncture or non-invasive sham control in a 1:1 ratio. Treatment will occur within 2 h before the operation, immediately after the operation upon arrival in the recovery ward, and once daily for 3 days. The primary outcome is the pain numerical rating scale, which will be analyzed using the area under the curve. The secondary outcome measures include heart rate and blood pressure after the operation, sleep quality during the hospital stay (actigraph), quality of recovery, and the 36-item short form health survey. This trial will use an intention-to-treat analysis.

**Discussion:**

This pilot randomized controlled trial will explore the feasibility of the further clinical application of electroacupuncture for the management of postoperative pain. It will inform the design of a further full-scale trial.

**Trial registration:**

Chinese Clinical Trial Registry, ChiCTR1900024183. Registered on 29 June 2019.

## Background

Postoperative pain is an important symptom following nasal endoscopic surgery for sinusitis due to nasal polyps. Effective management of postoperative pain after nasal endoscopic surgery is especially important for patient recovery. Pain due to a nasal filling may interfere with breathing, sleep quality, and patient satisfaction. According to previous reports, a failure to treat acute pain immediately may cause long-term chronic pain [1, 2]. The effective relief of acute pain is a challenge for patients and physicians following nasal endoscopy. Analgesic drugs, especially opioid analgesics, are often used to relieve pain after surgery. However, there are risks due to side effects, such as respiratory depression and drowsiness, which can lead to a delayed postoperative recovery. Thus, a safe and effective way to control postoperative pain and reduce recovery times needs to be identified.

Acupuncture has been widely used for pain relief, and its analgesic effects have been reported worldwide. Several studies have shown that acupuncture can relieve pain after surgery, including oral surgery [3, 4], cardiac surgery [5], laparoscopic cholecystectomy [6–8], gynecological surgery [9, 10], back surgery [11], and nasal surgery [12]. Therefore, acupuncture may relieve postoperative pain following nasal endoscopic surgery. Despite the positive results of these previously published studies, the results are not dependable due to issues such as small sample sizes, inadequate randomization, and lack of blinding. Therefore, high-quality evidence is needed to confirm the previous findings and for a protocol to be developed for the use of acupuncture for postoperative pain.

For those reasons, we designed a randomized controlled trial (RCT) using appropriate randomization, rigorous concealment of allocation, and blinding to examine the efficacy of electroacupuncture (EA) in relieving postoperative pain after nasal endoscopy. We aim to determine: (1) whether EA is useful for relieving postoperative pain after nasal endoscopy and (2) whether it can reduce postoperative recovery times. This study may provide high-quality evidence to support a standard protocol for the clinical application of EA for treating postoperative pain after nasal endoscopic surgery.

We hypothesize that EA is effective, safe, and feasible for postoperative pain management after nasal endoscopic surgery.

## Methods/design

### Study design

**Fig. 1:**
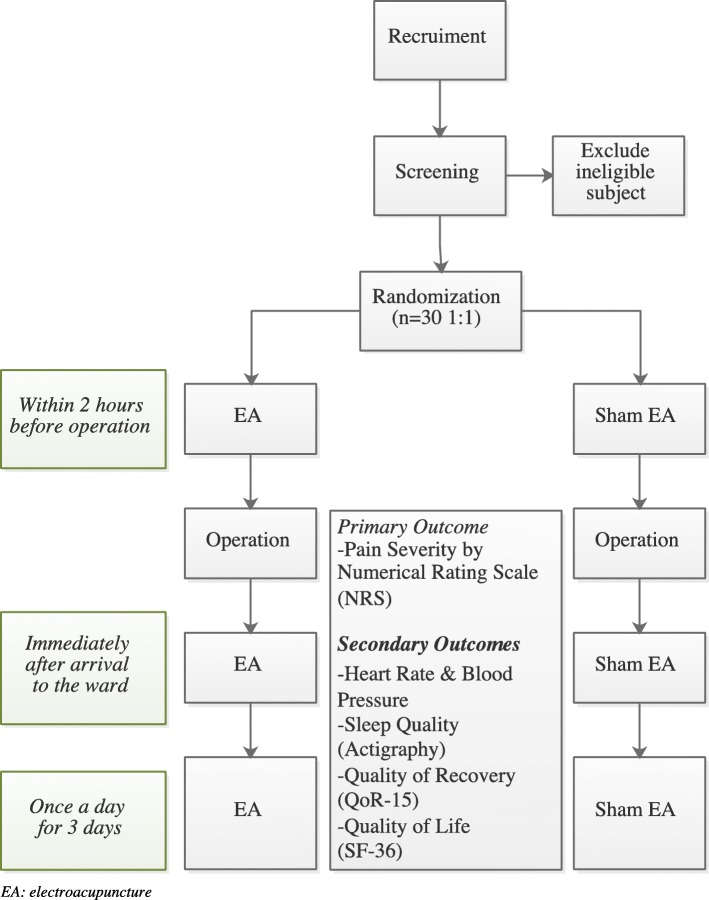
Flowchart for this study. EA electroacupuncture, QoR-15 15-item Quality of Recovery Scale, SF-36 36-item Short Form Health Survey

This is a single-site patient- and assessor-blinded placebo-controlled pilot RCT. It will be carried out in Shanghai Municipal Hospital of Traditional Chinese Medicine. Eligible patients will be randomly assigned to the EA group or the sham electroacupuncture (SEA) group in a 1:1 allocation ratio. All patients must sign the informed consent form before being enrolled in the trial. A flowchart of the study process is detailed in Fig. 1. Additional file 1 contains the completed Spirit checklist.

### Sample size

This is a pilot study. In the relevant literature, there are no previous studies utilizing the same evaluation method as the primary outcome to compare a sham acupuncture control. Therefore, we were unable to calculate the statistical power formally. The appropriate sample size for a two-arm pilot study should be more than 12. We assumed a 20% dropout rate, so that we will recruit 15 participants into each group. We will, therefore, recruit a total of 30 individuals for this RCT [13–15].

### Recruitment

Patients will be recruited to this RCT in Shanghai Municipal Hospital of Traditional Chinese Medicine. Eligible subjects who have been scheduled for nasal endoscopic surgery for sinusitis due to nasal polyps will be invited to participate. They will be referred from an otorhinolaryngologist. A research assistant will screen them and obtain written informed consent. They will then be randomly allocated to either the EA group or the SEA group. The schedule of enrolment, intervention, and assessments is detailed in Table 1.

**Table 1:** Schedule of enrolment, intervention, and assessments

	S	B	O	Preoperative period	Postoperative period		
Timepoint				Within 2 h before operation	Upon recovery	Hourly for 6 h after surgery	Once daily until discharge
Basic information							
Informed consent	X						
Inclusion/exclusion	X	X					
Medical history	X						
Vital signs	X	X	X	X	X	X	X
Interventions							
EA				X	X		X
Sham EA				X	X		X
Assessments							
Primary outcome							
NRS					X	X	X
Secondary outcomes							
HR and BP			X	X	X	X	X
Actigraphy		X	X				X
QoR-15					X		X^*^
SF-36					X		X^*^
Others							
Adverse events			X	X	X	X	X
Patient satisfaction							X
Success of blinding							X

*EA* electroacupuncture, *S* screening, *B* baseline, *O* operation, *NRS* numerical rating scale, *HR* heart rate, *BP* blood pressure, *QoR-15* 15-item Quality of Recovery Scale, *SF-36* 36-item Short From health survey

* On day of discharge

#### Inclusion criteria

Patients who meet all of the following criteria can be included:

1. American Society of Anesthesiologists physical status I to II
2. Eligible for nasal endoscopic surgery for sinusitis due to nasal polyps
3. Aged 18–60 years
4. Capable of understanding the trial and providing responses about the outcome measurement
5. Agree to participate in the survey and sign a written informed consent form

#### Exclusion criteria

Patients who meet any of the following criteria will be excluded:

1. Having chronic pain currently requiring treatment by an opioid or nonsteroidal anti-inflammatory drug
2. Having a severe psychiatric disease or cognitive impairment and not able to understand the trial
3. Having hepatitis B, hepatitis C, HIV infection, or syphilis
4. Having a history of alcohol or drug abuse
5. Having any local or systemic infection
6. History of acupuncture treatment in the past 6 months

### Randomization and allocation concealment

A random sequence will be produced by block randomization using the software SPSS version 23.0 by an independent research assistant. After the participants have completed the screening process and baseline assessment, they will be randomly assigned to one of the groups in a 1:1 ratio using random numbers in opaque envelopes. The treatment allocation codes will not be revealed before the first treatment. In an effort to minimize breaks in coding, the principal investigator who designed the trial and the research personnel who perform the outcome assessments will also be blinded to the treatment assignment.

Before the beginning of treatment, we will tell the participants that they have the same probability of being assigned to the EA group or the SEA group. To ensure that participants remain blinded, they will wear an eye mask during treatment. Only the acupuncturist who performs the treatment will know the group allocation at the time of treatment. The principal investigator, the data analysts, the outcome assessors, and statistician will remain blinded.

### Quality control

The assessors and acupuncturists will be trained before the trial by the principal investigator (SX) to ensure the quality of this trial. This training covers inclusion and exclusion criteria, location of the acupoints, and the depth of needling. We will perform the EA procedure following the guidance for the clinical practice of acupuncture [16]. The acupuncturists must be registered, have a master’s degree, and have at least 3 years of experience in practicing acupuncture. In addition, data management, collecting outcome measures, and the statistical analysis will be completed by three independent researchers.

### Intervention protocol

All participants will be asked to fast from 8 h before the operation. They will undergo a standard operative procedure and receive postoperative treatment. Participants will be transferred to the ward within 30 min after the operation. EA or SEA will be given within 2 h prior to the surgery, immediately after surgery on arrival in the recovery ward, and daily following surgery for 3 days (five treatment sessions in total). If a participant drops out before completing the study, the reason will be recorded.

The acupuncture methods are described in Table 2. In each treatment session, the patients will be in a private area and placed in the supine position. They will wear an eye mask to prevent them from observing the treatment procedure. The researcher will sterilize the patient’s skin with 75% alcohol wipes before treatment. Each treatment session will last for 30 min. When the needles are removed, the researcher will use a clean piece of cotton wool to prevent bleeding by compressing the points. In the first treatment, 11 acupoints will be used and 17 acupoints will be used in the subsequent postoperative treatments. The acupoints and rationale are summarized in Table 3.

**Table 2:** Treatment methods of electroacupuncture and acupoints

	EA group	SEA group
Acupoints	All sessions: LI4, PC6, HT7, LI20, ST36, GV23 Postoperatively: GV20, GV29, EX-HN22, SP6	All sessions: LI4, PC6, HT7, LI20, ST36, GV23 Postoperatively: GV20, GV29, EX-HN22, SP6
Depth of insertion	10 mm: HT7, GV20, GV23, GV29, LI4, LR3	No insertion
	30 mm: LI20, SP6, PC6, ST36	
Needle type	Steel needles (Wuxi Jiajian Medical Co. Ltd. Wuxi, China)	Blunt needles (Streitberger placebo needle)
Needle sensation	With *de-qi* sensation	Without *de-qi* sensation
Electric stimulation	Three pairs of needles: LI4–ST36 (bilaterally), GV20–GV23 Connected to an electric stimulator (HANS-200B) Deliver continuous wave-type mid-frequency (15Hz) current of 2 mA	Three pairs of needles: LI4–ST36 (bilaterally), GV20–GV23 Connected to an electric stimulator (HANS-200B) No electrical current delivered

*EA* electroacupuncture, *SEA* sham electroacupuncture

**Table 3:** Acupoint selection and rationale based on traditional Chinese medicine

Acupoint	Location	Traditional Chinese medicine indication	Suggested technique
LI4 (*hegu*)	Dorsum of hand, at the level of the midpoint of the second metacarpal bone, between the first and second metacarpal bones	Alleviates nasal pain	Needle perpendicular, 0.5–1.0 *cun*
PC6 (*neiguan*)	Palmar aspect of the forearm, between the tendons, 2 *cun* away from the transverse crease of the wrist	Alleviates gastric pain and calms the *shen (spirit)*	Needle perpendicular, 0.5–1.5 *cun*
HT7 (*shenmen*)	Palmar aspect of the wrist, ulnar end of the transverse crease	Helps in calming the *shen* (spirit)	Needle perpendicular, 0.3–0.5 *cun*
LI20 (*yingxiang*)	0.5 *cun* lateral to the midpoint of the border of line ala nasi, in the nasolabial groove	Helps in nasal congestion and epistaxis.	Needle perpendicular, 0.3–0.5 *cun*
ST36 (*zusanli*)	Antero-lateral leg, 1 middle-finger breadth next to the anterior crest of tibia, 3 *cun* under the depression lateral to the patellar ligament	Helps in regulating *qi* and blood circulation	Needle perpendicular, 0.5–1.5 *cun*
GV23 (*shangxing*)	1 *cun* directly above the midpoint of the anterior hairline	Helps in rhinorrhea, epistaxis.	Needle perpendicular, 0.5–1.0 *cun*
GV20 (*baihui*)	5 *cun* directly above the midpoint of the anterior hairline, at the midpoint of the line connecting the apexes of the two auricles	Helps in calming the Shen (spirit)	Needle perpendicular, 0.5–0.8 *cun* Moxibustion can be used to reinforce the *yang*
GV29 (*yintang*)	On the forehead, at the midpoint between the two medial ends of the eyebrow	Helps in insomnia, dizziness; epistaxis, rhinorrhea.	Needle perpendicular, 0.3–0.5 *cun*
EX-HN22 (*anmian*)	On the napex, at the midpoint between TE17 and GB20	Helps in calming the Shen (spirit), improving insomnia.	Needle perpendicular, 0.8–1.2 *cun* Moxibustion can be used to reinforce the *yang*
SP6 (*sanyinjiao*)	Behind the medial tibia, 3 *cun* above the tip of the medial malleolus	Helps in regulating Qi and blood circulation improving insomnia, dizziness.	Needle perpendicular, 0.5–1.0 *cun*

### The electroacupuncture group

The sterile disposable acupuncture needles are made of standard stainless steel (0.25 × 40 mm and 0.30 × 40 mm; Jia Jian, China). In all sessions, patients will receive treatment at 11 standard acupuncture points: bilateral *hegu* (LI4), *neiguan* (PC6), *shenmen* (HT7), *yingxiang* (LI20), *zusanli* (ST36), and *shangxing* (GV23). The postoperative treatment will be the same as the preoperative EA treatment, except that we will add the following six points: *baihui* (GV20), *yintang* (GV29), *anmian* (EX-HN22), and *sanyinjiao* (SP6). All acupoints were selected according to the acupuncture textbook of the International Standard Library of Chinese Medicine written by Zhang, Zhao, and Lao [17], the literature, and our clinical experience. The acupoints will be located with reference to the textbook. First the researcher will manipulate the needle manually until the patient reports needling sensations (*de-qi* sensation). An electric stimulator (HANS-200B) will be connected to three pairs of needles (LI4–ST36 bilaterally and GV20–GV23) to deliver a continuous wave-type low-frequency (2 Hz) current of 2 mA.

### Control group

In the control group, Streitberger placebo needles, a sham acupuncture device that has been widely reported and validated, will be used [18]. The non-invasive sham needles will be positioned 1.5 *cun* lateral and posterior to the true acupoints [19, 20]. The blunt tip of the sham needles will touch the surface of the skin but will not penetrate it. The electric stimulator (HANS-200B) will be beside the patients and three pairs of electrodes will be connected to LI4–ST36 (bilaterally) and GV20–GV23 for 30 min but no electrical current will be delivered.

### Outcome measures

The participant’s recovery will be monitored during and after the operation. The primary outcome is postoperative pain scores measured using a NRS. The secondary outcomes include heart rate (HR) and blood pressure (BP) after the operation, sleep quality during the hospital stay (actigraphy), quality of recovery, and overall health. In addition, the exact type of operation and when EA or SEA treatment is given will be recorded for analysis. If the participants cannot be managed according to the protocol for any reason, including side effects, no further data will be collected. The assessment schedule is detailed in Table 1.

#### Primary outcome

NRS is a commonly used scale for assessing clinical pain. It is easier for patients to grade pain intensity with numbers rather than other measurements like the visual analog scale. The NRS is an 11-point scale for pain, with 0 indicating no pain and 10 indicating the worst possible pain. NRS pain scores will be assessed hourly for 6 h after surgery, then daily for 3 days [21, 22].

#### Heart rate and blood pressure

HR and BP will be monitored during and after the operation. These vital signs have been extensively utilized and validated. HR and BP will be assessed hourly for 6 h after surgery, then daily for 3 days.

#### Sleep quality

An actigraph device measures sleep quality when worn on the wrist overnight. The main sleep indexes are sleep efficiency, total sleep time, and sleep awakenings. Sleep condition and sleep quality will be analyzed using the software ActiLife6 (Version 6.8.1, Actigraph LLC) [23]. Sleep quality will be assessed five times: the night before the operation, the night after the operation, and three subsequent nights until discharge.

#### Quality of recovery

The 15-item Quality of Recovery Scale (QoR-15) is a valid, extensive, and efficient evaluation of a patient’s postoperative recovery. The QoR-15 measures five aspects of recovery: pain, physical comfort, physical independence, psychological support, and emotional state. The questionnaire consists of 15 items with a score from 0 to 10, with 0 representing no pain and 10 representing pain always present. The sum of the scores is the patient’s QoR-15 score [24]. QoR-15 will be assessed twice: after surgery and on the day of discharge.

#### Overall health

The 36-item Short Form Health Survey (SF-36) consists of 36 items in eight multi-item scales (physical functioning, physical role, bodily pain, general health, vitality, social functioning, emotional role, and mental health) and one single-item scale (health transition). Higher scores represent better health status [25]. SF-36 will be assessed twice: after surgery and on the day of discharge.

### Safety assessment

Before signing the informed consent form, participants will be told about the potential adverse events related to acupuncture, such as bruises, hematomas, infection, and pain. Adverse events will be graded as 1 (mild), 2 (moderate), or 3 (severe or medically significant). Any adverse events that occur during the trial will be recorded by the patients and doctors. Vital signs will be assessed at the time of the event, including HR, BP, and respiratory rate. All details of adverse events will be reported in a case report form. If any severe adverse event occurs, we will terminate the EA or SEA treatment and the Clinical Research Center for Drugs of the Shanghai University of Traditional Chinese Medicine will unblind the participant to determine whether the serious adverse event is related to EA or SEA. A final decision will made on whether to continue the study. We will analyze the influence of all events at the end of the trial.

### Credibility of Treatment Rating Scale

We will use the Credibility of Treatment Rating Scale to assess, from the participants’ viewpoint, the credibility of the acupuncture treatments [26, 27]. This scale has four items: (1) the perceived logic of the treatment, (2) whether they would recommend the treatment to their friends who have similar complaints, (3) whether they believe that the treatment will alleviate their complaint, and (4) whether they believe that the treatment will alleviate other complaints.

### Blinding success assessment

Once the final treatment has been completed, we will test the success of blinding by asking the participants the following question: “When you volunteered for the study, you were informed that you had equal odds of receiving traditional electroacupuncture or electroacupuncture-like treatment. Now that our study is complete, which type of treatment do you think you received?” Three choices will be provided for participants: EA group, SEA group, and uncertain. If participants do not choose “uncertain,” we will ask them why they made their choice [3].

### Monitoring

To ensure the quality of this trial, the whole process will be supervised by a qualified clinical trial expert. The trial will be carried out by Shanghai Municipal Hospital of Traditional Chinese Medicine. The Clinical Research Center for Drugs of the Shanghai University of Traditional Chinese Medicine will monitor the data, including interim results. If any problems are identified, the center may decide to change this protocol. Any such changes will be announced to all persons involved in the trial in writing after they have been approved by the relevant ethics committee. In addition, a qualified clinical trial expert will monitor this study. The principal investigator will take full responsibility for the conduct of the trial and will make the final decision on any changes required.

### Data management

The clinical trial management platform ResMan will be used to manage the original data, which will be collected by blinded assessors and double-entered. The data management system will be tested beforehand, and relevant users will be trained on it before it is officially launched. The database will be protected by passwords. The original data for a participant will be entered within 1 week after their discharge from hospital. If there are issues with the data, the data supervisor will request clarification from a researcher. The clinical supervisor will monitor the work of the clinical trial center at least once a month.

### Statistical analysis

An independent statistician blinded to group allocation will perform the statistical analyses using the statistical software SPSS 23.0 for Windows. The analyses will include an intention-to-treat analysis incorporating data from any participants who have dropped out of the trial. We will use multiple imputation for missing data. All demographic and clinical characteristics of the subjects (such as sex, age, and weight) will be analyzed using descriptive statistics. We will identify the homogeneity of demographic characteristics and study variables between the two groups. Continuous variables will be reported as means ± standard deviations whereas qualitative data will be presented as frequencies and percentages. To analyze the primary outcome, the area under the curve of the NRS pain scores will be calculated using the trapezoidal method. Comparisons between groups will be made using Student’s *t*-test. The secondary outcomes—BP, HR, QoR-15, SF-36, and actigraphy assessments (total sleep time, sleep awakenings, and sleep efficiency)—will be compared between the two groups with Student’s *t*-test or the Wilcoxon rank-sum test. All reported *p* values will be two-sided, and *p* < 0.05 is considered statistically significant.

## Discussion

Postoperative pain is common after nasal endoscopic surgery. It can interfere with the quality of sleep and delay postoperative recovery. It is also a significant burden on individuals. We chose to study acupuncture because previous studies have shown that it is an effective and safe method of pain management [11, 28–30]. If postoperative pain is not treated immediately, patients can develop long-term chronic pain. However, EA specifically for the relief of postoperative pain after nasal endoscopic surgery has not yet been studied through a RCT.

This study will be conducted at the Shanghai Municipal Hospital of Traditional Chinese Medicine. Acupuncture has already been used for inpatients. However, it has not been used for immediate analgesia for a surgical patient. This study is designed to investigate the efficacy and safety of acupuncture as an adjunctive therapy for relieving postoperative pain and discomfort. If this study confirms that acupuncture is effective and safe, acupuncture could be implemented as a new standard for relieving postoperative pain in Chinese and Western medicine.

There are several challenges and limitations in this study. Firstly, patients with different types of nasal disease will be screened to reduce variability within the study population. Therefore, only participants who need nasal endoscopic surgery for sinusitis due to nasal polyps will be included. In addition, randomization will be done rigorously so that the number of participants in the two groups is balanced. Secondly, the preoperative EA or SEA treatment must be carried out in the recovery ward within 2 h before the operation. We found many studies in which the acupuncture was performed before the induction of anesthesia in the operating theater [6, 31, 32]. However, the surgical team were concerned that this would lengthen the pre-surgical preparation time. Therefore, the timing of the preoperative treatment may not be ideal. Thirdly, it is difficult to schedule the acupuncture treatment because the surgery time is uncertain. An acupuncturist must wait until the surgery has ended so that they can immediately perform the postoperative treatment. Fourthly, because of the nature of clinical trials of acupuncture, it is inevitable that the acupuncturist is aware of treatment allocation. However, the acupuncturists will not be involved in assessing patients or the analysis. To prevent an acupuncturist from accidentally revealing the group allocation, their interactions with the patients will be limited. All patients will wear an eye mask while receiving treatment, which will occur in a private area. Overall, blinding will be strictly enforced.

Though there are many difficulties, we will strive to standardize the steps of the study to ensure its quality. We hope this pilot RCT will provide clinical evidence for the feasibility of EA in alleviating postoperative pain. The outcomes will inform the design of a further full-scale trial.

### Trial status

The first investigators’ meeting took place on 31 May 2019. The RCT is in preparation now and will launch on 1 May 2020. Recruitment is expected to end in late 2020.

## Supplementary information

**Additional file 1:** SPIRIT 2013 checklist.

## Data Availability

The trial results will be published in peer-reviewed scientific papers and in posters or oral presentations in conferences. All data and the protocol will be available 3 months after our publication of the results for a period of 3 years. The trial data will be available from the corresponding author upon reasonable request.

## Abbreviations

BP: Blood pressure
EA: Electroacupuncture
HIV: Human immunodeficiency virus
HR: Heart rate
NRS: Numerical rating scale
QoR-15: 15-item Quality of Recovery Scale
RCT: Randomized controlled trial
SEA: Sham electroacupuncture
SF-36: 36-item Short Form Health Survey
